# Network analysis of plasma proteomes in affective disorders

**DOI:** 10.1038/s41398-023-02485-4

**Published:** 2023-06-09

**Authors:** Sang Jin Rhee, Dongyoon Shin, Daun Shin, Yoojin Song, Eun-Jeong Joo, Hee Yeon Jung, Sungwon Roh, Sang-Hyuk Lee, Hyeyoung Kim, Minji Bang, Kyu Young Lee, Jihyeon Lee, Jaenyeon Kim, Yeongshin Kim, Youngsoo Kim, Yong Min Ahn

**Affiliations:** 1 Biomedical Research Institute, Seoul National University Hospital, Seoul, Republic of Korea; 2 Department of Biomedical Science, School of Medicine, CHA University, Seongnam, Republic of Korea; 3 Department of Psychiatry, Seoul National University College of Medicine, Seoul, Republic of Korea; 4 Department of Neuropsychiatry, Seoul National University Hospital, Seoul, Republic of Korea; 5 Department of Neuropsychiatry, School of Medicine, Eulji University, Daejeon, Republic of Korea; 6 Department of Psychiatry, Uijeongbu Eulji Medical Center, Eulji University, Uijeongbu, Republic of Korea; 7 Department of Psychiatry, SMG-SNU Boramae Medical Center, Seoul, Republic of Korea; 8 Institute of Human Behavioral Medicine, Seoul National University Medical Research Center, Seoul, Republic of Korea; 9 Department of Psychiatry, Hanyang University Hospital and Hanyang University College of Medicine, Seoul, Republic of Korea; 10 Department of Psychiatry, CHA Bundang Medical Center, CHA University School of Medicine, Seongnam, Republic of Korea; 11 Department of Psychiatry, Inha University Hospital, Incheon, Republic of Korea; 12 Department of Psychiatry, Nowon Eulji University Hospital, Seoul, Republic of Korea; 13 Department of Biomedical Sciences, Seoul National University College of Medicine, Seoul, Republic of Korea

**Keywords:** Molecular neuroscience, Physiology

## Abstract

The conventional differentiation of affective disorders into major depressive disorder (MDD) and bipolar disorder (BD) has insufficient biological evidence. Utilizing multiple proteins quantified in plasma may provide critical insight into these limitations. In this study, the plasma proteomes of 299 patients with MDD or BD (aged 19–65 years old) were quantified using multiple reaction monitoring. Based on 420 protein expression levels, a weighted correlation network analysis was performed. Significant clinical traits with protein modules were determined using correlation analysis. Top hub proteins were determined using intermodular connectivity, and significant functional pathways were identified. Weighted correlation network analysis revealed six protein modules. The eigenprotein of a protein module with 68 proteins, including complement components as hub proteins, was associated with the total Childhood Trauma Questionnaire score (*r* = −0.15, *p* = 0.009). Another eigenprotein of a protein module of 100 proteins, including apolipoproteins as hub proteins, was associated with the overeating item of the Symptom Checklist-90-Revised (*r* = 0.16, *p* = 0.006). Functional analysis revealed immune responses and lipid metabolism as significant pathways for each module, respectively. No significant protein module was associated with the differentiation between MDD and BD. In conclusion, childhood trauma and overeating symptoms were significantly associated with plasma protein networks and should be considered important endophenotypes in affective disorders.

## Introduction

The conventional differentiation of affective disorders into major depressive disorder (MDD) and bipolar disorder (BD) is based on the history of (hypo)manic symptoms [1]. As treatment regimens and outcomes differ between these disorders, there has been considerable effort to differentiate these disorders, including the use of biological correlates [2, 3]. Top-down biological approaches have expanded our knowledge to facilitate the differentiation of these disorders. However, there are limitations with regard to inconsistency and modest accuracy [4, 5]. Understanding affective disorders based on biological correlates with a transdiagnostic bottom-up approach may explain these limitations and deepen our knowledge of the pathophysiology of these disorders.

Proteomics-based research has received growing interest as proteomes reflect biological functions [6]. Recent technological advances have enabled researchers to simultaneously quantify multiple proteins [7]. While previous studies relied on few markers, multiplexing now permits the construction of networks between multiple proteins [8]. These approaches have focused on comparing specific diseases with healthy controls. For instance, a study from the NESDA (Netherlands Study of Depression and Anxiety) constructed networks with 171 blood proteomes to explain the differentiation between MDD and healthy controls [9]. However, to our knowledge, no study to date has applied this approach trans-diagnostically in individuals with affective disorders.

In this study, we implemented weighted correlation network analysis to identify biologically meaningful modules of interconnected proteins in plasma samples from individuals with affective disorders, including both MDD and BD. Further analysis was performed to determine meaningful traits associated with these modules and to identify hub proteins from these modules.

## Materials and methods

### Clinical samples

The initial study population comprised 169 patients with MDD and 141 patients with BD from our previous study [10]. In total, 26 patients with BD with a Young Mania Rating Scale (YMRS) total score >12 had been excluded to rule out those with current (hypo)manic symptoms, and 8 patients additionally had been excluded due to missing data of covariates. Patients were enrolled between August 2018 and December 2020 from 6 hospitals, including Seoul National University Hospital (SNUH); Nowon Eulji Medical Center, Eulji University; Seoul Metropolitan Government Seoul National University Boramae Medical Center; Hanyang University Hospital; Inha University Hospital; and Cha University Bundang Medical Center. The diagnosis was based on the Diagnostic and Statistical Manual of Mental Disorders, Fifth Edition (DSM-5) and was confirmed with the Mini-International Neuropsychiatric Interview (MINI). Only patients with Clinical Global Index—Severity (CGI-S) ≥ 3 were included.

Patients were excluded if they had taken any anti-inflammatory analgesic within the 2 preceding weeks; had a history of neurosurgery, central nervous system (CNS) diseases, cancer, and tuberculosis; had a substance use disorder other than alcohol, caffeine, and nicotine; were currently lactating or pregnant; and/or were predicted to have an intellectual disability or difficulty in understanding Korean. These exclusions were predominantly based on previous evidence of known associations between these conditions and protein expression [11*–*19]. Patients with a history of neuromodulation or intensive psychotherapy for the past 2 months were also excluded to confine the effect of treatment to psychotropic medications.

Plasma samples from each individual were collected in a 6-mL ethylenediaminetetraacetic acid (EDTA) tube (ref. 367863, Becton, Dickinson and Company, Trenton, NJ) and centrifuged at 1100–1300*g* for 10–15 min at 4 °C or room temperature. The collected supernatant was stored in Eppendorf tubes at ≤−70 °C until usage.

The authors assert that all procedures contributing to this work comply with the ethical standards of the relevant national and institutional committees on human experimentation and with the Helsinki Declaration of 1975, as revised in 2008. Informed consent for each participant was obtained. The study was reviewed by the Institutional Review Boards of Seoul National University Hospital (IRB No. 1806-1065-951) and all other participating hospitals.

### Demographics and clinical features

The demographics considered in this study were age, sex, body mass index (BMI), blood collection time, fasting time, current alcohol use, exercise status, and smoking status [10]. Both age and BMI were analyzed as continuous variables. Sex (male/female), blood collection time (AM, PM), fasting time (<8 h, ≥8 h), current alcohol use, exercise status, and smoking status (yes/no) were analyzed as dichotomous variables. Alcohol use was defined as at least one drink once per week. Exercise status was classified based on the World Health Organization (WHO) recommendations for moderate-intensity physical activity for at least 30 min once per week [20].

Medication usage was classified as a dichotomous variable for antipsychotics, lithium/anticonvulsants, antidepressants, and benzodiazepines/hypnotics. Disease chronicity was assessed with the duration from first onset and duration from first medication (years).

Objective symptom severity was assessed with the Brief Psychiatric Rating Scale (BPRS) [21], YMRS [22], Montgomery-Asberg Depression Rating Scale (MADRS) [23], and Hamilton Anxiety Scale (HAMA) [24]. We conducted subjective self-reports, including the Symptom Checklist-90-Revised (SCL-90-R) [25], brief form of the World Health Organization Quality of Life Assessment Instrument (WHOQOL-BREF) [26], Childhood Trauma Questionnaire (CTQ) [27], short form of the Wender-Utah Rating Scale (WURS) [28], Composite Scale of Morningness (CSM) [29], and Seasonal Pattern Assessment Questionnaire (SPAQ) [30].

### Plasma proteomic quantification

Specific methods for the following analysis have been described in our previous study [10]. In brief, for 44 μL of each plasma sample, the six highest abundant proteins were depleted with the MARS-6 column (Agilent Technologies, Santa Clara, CA, USA). In total, 100 μg of quantified protein was reduced with 40 μL of 0.2% RapiGest solution and 20 mM dithiothreitol (DTT) at 60 °C for 1 h and then alkylated with 20 μL of 100 mM iodoactamide (IAA) in the dark at room temperature for 30 min. Then, the samples were digested with trypsin solution at 37 °C for 4 h, and the digestion was completed by adding 10% formic acid. The sample was centrifuged at 15,000 rpm for 1 h at 4 °C, and the supernatants were spiked with crude stable isotope-labeled internal standard (SIS) peptides, in which a C-terminal lysine or arginine was heavy isotope-labeled (^13^C_6_^15^N_2_ or ^13^C_6_^15^N_4_) (purity: crude (>70%), JPT, Berlin, Germany). There were 5 preparation batches, and for each batch, the samples were randomly distributed and assigned identification numbers to blind the researchers throughout the sample preparation. From reviewing databases of psychiatric disorders, established targets for affective disorders, and laboratory-established targets, and after checking for detectability and quantifiability, 642 target peptides were initially selected [10].

Liquid chromatography–multiple reaction monitoring–mass spectrometry (LC–MRM–MS) was performed with a 1260 Infinity HPLC system equipped with a Jetstream electrospray source coupled to an Agilent 6490 triple quadrupole MS (Agilent Technologies, Santa Clara, CA, USA). The sample vials of the autosampler were maintained at 4 °C, and the guard/analytical columns were maintained at 40 °C. For each digested sample, 40 μL was injected into a guard column (2.1 × 15.0 mm, 1.8 µm, 80 Å) (Agilent Technologies, Santa Clara, CA, USA). Online desalting was conducted in 3% solvent B (formic acid/acetonitrile (v/v)) at 50 μL/min for 10 min. After the valve position was switched, the sample was transferred to an analytical column (0.5 × 35.0 mm, 3.5 µm, 80 Å) (Agilent Technologies, Santa Clara, CA, USA) in 3% solvent B at 40 µL/min for 5 min. Bound peptides were separated on the column and eluted with a linear gradient of 3% to 35% solvent B at 40 µL/min for 50 min.

Mass spectra were generated in positive ion mode, with the following parameters: 2500 V ion spray capillary voltage, 2000 V nozzle voltage, 5 V cell accelerator voltage, 200 V delta EMV, and 380 V fragmented voltage. The drying gas was sprayed at 15 L/min at 250 °C, and the sheath gas flow was 12 L/min at 350 °C. The collision energy was optimized by adding the intensities of individual transitions that resulted in the largest peak area. SIS peptides were first pooled and analyzed to evaluate their retention times. The retention times were compared with those of endogenous peptides by analyzing the matrix of endogenous peptides with SIS peptides and a heavy β-galactosidase peptide. Subsequently, the final targets in individual blood samples were quantified. LC–MRM–MS analysis was performed once per sample (1 replicate for each sample).

Raw data from the LC–MRM–MS analysis was processed in Skyline (version 19.1.0) (MacCoss Lab, Seattle, WA, USA). Peptide quantification was calculated with the peak area ratio (PAR), defined as the ratio of endogenous to SIS peptide peak area. From 642 target peptides, 54 unstable peptides with low intensities (intensity < 1000), unequal retention times between light and heavy peptides, and skewed peaks were excluded. Subsequently, the final PAR values of 588 target peptides were normalized to the area of heavy β-galactosidase peptide.

### Weighted correlation network analysis

An overview of the analysis is presented in Supplementary Fig. 1. Peptides with PAR values ≤ 0.01 or ≥100 for at least 5% of the final study population were initially excluded, in accordance with our previous study [10]. For proteins with multiple peptides quantified, the mean value was used for further analysis, resulting in a total of 420 proteins. Weighted correlation network analysis was conducted with the WGCNA package in R [31]. Initially, 8 samples were excluded as outliers by hierarchical clustering. Logarithmic transformation followed by batch correction was performed with the Combat algorithm for preparation batches presuming effects of age, sex, and BMI. Linear regression was performed to regress out the effects of age, sex, and BMI, and the residuals were used for further analysis. Three other samples were excluded as outliers by hierarchical clustering after preprocessing. A signed, weighted network was constructed [8] based on Pearson’s correlation and was converted with the smallest soft thresholding power to generate a scale-free network (scale-free *R*^2^ ≥ 0.9). After calculating a topological overlap measure (TOM) based on the adjacency matrix, a TOM dissimilarity matrix (1-TOM) was used to perform hierarchical clustering and map a dendrogram. Proteins were divided into different modules using the dynamic tree cut algorithm with the following parameters: deepSplit = 4, minimal module size = 20. Each module was summarized by the first principal component of protein expression across individuals, referred to as the module eigenprotein. Modules were merged if the difference between their module eigenprotein profiles was <0.25. The correlations between modules (represented with the module eigenprotein) and demographic/clinical traits were analyzed with Pearson correlation analysis. The association of modules with hospital type was assessed using ANOVA. Additional analysis to control for significant demographic traits when comparing the association between modules and clinical traits was performed using linear regression.

Module membership was defined as the correlation between protein expression profiles and the module eigenprotein [8]. For each protein, a protein significance measure was estimated as the absolute value of correlation between the expression profile and specific traits to identify proteins strongly associated with the traits [8]. The correlation between module membership and protein significance was analyzed to examine the presence of a linear relationship. Additionally, the network results were imported to Cytoscape software (Version 3.9.1) to select the top 10 hub proteins (intramodular-connected). This was performed based on the maximal clique centrality (MCC) with the Cytohubba (Version 0.1) plug-in [32, 33].

Proteins from significant modules were entered in the DAVID bioinformatics resource tool (https://david.ncifcrf.gov/) for gene ontology analysis with the official gene names [34]. The gene ontology classification for biological processes, cellular components, and molecular functions was conducted using Fisher’s exact test, with a cut-off value of 0.05. Additionally, pathway analysis was performed using the KEGG (Kyoto Encyclopedia of Genes and Genomes) database (http://www.genome.jp/kegg/) [35].

Finally, network stability was assessed by iterating network reconstruction using the same settings while only including 63% of randomly sampled patients from the final study population, which was repeated 100 times with the *sampledBlockwiseModules* function. For each protein, consistency was calculated as the percentage of the 100 sub-samplings in which the protein was assigned to the same module. The stability of each module was defined as the average consistency of all proteins in the given module.

### Statistical analysis

Statistical analysis of demographic/clinical characteristics and targeted proteomic data was performed with SPSS version 21.0 and R version 4.1.2. Statistical tests were two-tailed. *P*-values < 0.05 were considered statistically significant. For functional analysis, the Benjamini-FDR corrected *q*-value was calculated.

## Results

### Demographics and clinical characteristics of the final population

The demographic and clinical characteristics of the study population after excluding outliers are presented in Table 1. The average age was 35.22 ± 13.01 years, 33.4% were male, and 45.2% were diagnosed with BD. The mean values of total MADRS and YMRS scores were 22.55 ± 10.98 and 2.48 ± 3.07, respectively.

**Table 1:** Demographic and clinical characteristics of the final study population (*n* = 299).

Age, mean ± SD, years	35.22 ± 13.01
Sex (Male), *n* (%)	100 (33.4%)
BMI, mean ± SD, kg/m^2^	23.84 ± 4.20
Blood collection time: AM, *n* (%)	91 (30.4%)
Fasting time: at least 8 h, *n* (%)	68 (22.7%)
Alcohol drinking (at least once a week), *n* (%)	107 (35.8%)
Exercise (moderate), *n* (%)	98 (32.8%)
Current smoker, *n* (%)	103 (34.4%)
Antipsychotics, *n* (%)	166 (55.5%)
Lithium/Anticonvulsants, *n* (%)	118 (39.5%)
Antidepressants, *n* (%)	178 (59.5%)
Benzodiazepine/hypnotics, *n* (%)	190 (63.5%)
Psychiatric disorders	
Bipolar disorder, *n* (%)	135 (45.2%)
Major depressive disorder, *n* (%)	164 (54.8%)
Duration of the first onset, mean ± SD, years	7.86 ± 8.38
Duration of the first medication, mean ± SD, years	5.13 ± 7.38
BPRS, mean ± SD	39.62 ± 7.24
YMRS, mean ± SD	2.48 ± 3.07
MADRS, mean ± SD	22.55 ± 10.98
HAM-A, mean ± SD	12.78 ± 7.06
SCL-90-R	
Somatization dimension, mean ± SD	1.14 ± 0.89
Obsessive–compulsive dimension, mean ± SD	1.69 ± 0.87
Interpersonal sensitivity dimension, mean ± SD	1.45 ± 0.88
Depression dimension, mean ± SD	1.95 ± 0.97
Anxiety dimension, mean ± SD	1.39 ± 0.94
Hostility dimension, mean ± SD	1.02 ± 0.91
Phobic anxiety dimension, mean ± SD	0.92 ± 0.90
Paranoid ideation dimension, mean ± SD	1.02 ± 0.87
Psychoticism dimension, mean ± SD	1.08 ± 0.77
Overeating item, mean ± SD	1.12 ± 1.29
WHOQOL-BREF, mean ± SD	65.91 ± 12.63
CTQ, mean ± SD	51.88 ± 16.82
WURS, mean ± SD	30.72 ± 21.31
CSM, mean ± SD	29.71 ± 8.01
SPAQ, mean ± SD	5.59 ± 5.37

*SD* standard distribution, *BMI* body mass index, *BPRS* Brief Psychiatric Rating Scale, *YMRS* Young Mania Rating Scale, *MADRS* Montgomery-Asberg Depression Rating Scale, *HAM-A* Hamilton Anxiety Scale, *SCL-90-R* Symptom Checklist-90-Revised, *WHOQOL-BREF* brief form of the World Health Organization Quality of Life Assessment Instrument, *CTQ* Childhood Trauma Questionnaire, *WURS* short form of the Wender-Utah Rating Scale, *CSM* Composite Scale of Morningness, *SPAQ* Seasonal Pattern Assessment Questionnaire.

### Weighted correlation network analysis

**Fig. 1:**
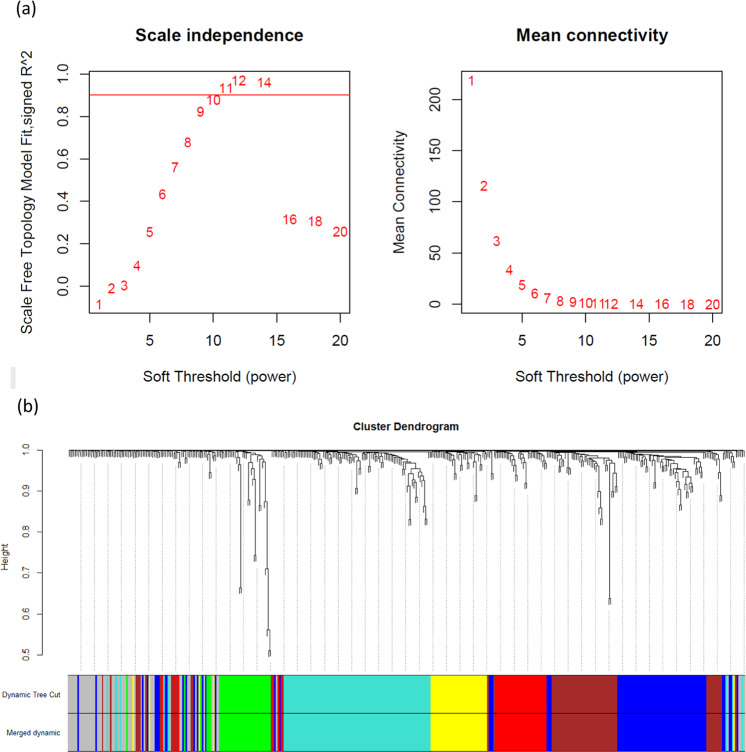
Protein co-expression network construction. **a** Analysis of the scale-free fit index (left) and mean connectivity (right) as a function of soft-threshold powers. **b** Clustering dendrograms of the proteins with the topological overlap measure dissimilarity matrix, with assigned module colors by the initial dynamic tree cut and the final merged dynamic, which were identical.

**Fig. 2:**
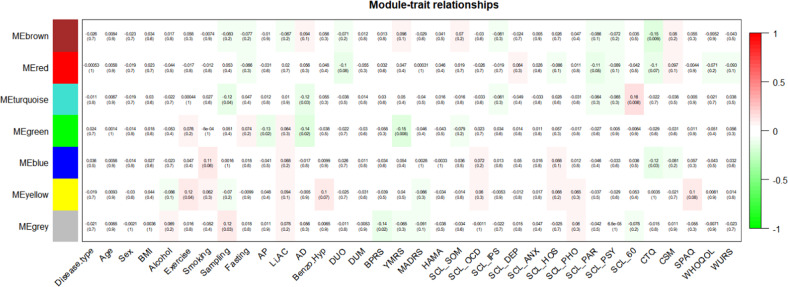
Heat map representation of module-trait relationships. Each row represents a module eigenprotein, and each column represents demographic/clinical traits. Each cell contains Pearson’s correlation and *p*-values. The color is coded by correlation with the legend on the right. Disease.type major depressive disorder versus bipolar disorder, BMI body mass index, AP antipsychotics use, Li.AC lithium/anticonvulsants use, AD antidepressants use, Benzo.Hyp benzodiazepine/hypnotics use, DUO duration of first onset, DUM duration of first medication, BRPS Brief Psychiatric Rating Scale, YMRS Young Mania Rating Scale, MADRS Montgomery-Asberg Depression Rating Scale, HAMA Hamilton Anxiety Scale, SCL Symptom Checklist-90-Revised, SOM somatization dimension, OCD obsessive–compulsive dimension, IPS interpersonal sensitivity dimension, DEP depression dimension, ANX anxiety dimension, HOS hostility dimension, PHO phobic anxiety dimension, PAR paranoid ideation dimension, PSY psychoticism dimension, 60 overeating item, CTQ Childhood Trauma Questionnaire, CSM Composite Scale of Morningness, SPAQ Seasonal Pattern Assessment Questionnaire, WHOQOL-BREF brief form of the World Health Organization Quality of Life Assessment Instrument, WURS short form of the Wender-Utah Rating Scale.

A signed weighted network was constructed based on Pearson’s correlation with soft thresholding power = 11 for a scale-free network (scale-free *R*^2^ ≥ 0.9) (Fig. 1a). In total, 6 protein modules were identified (excluding the gray module), and no modules needed to be merged (Fig. 1b, Supplementary Fig. 2, Supplementary Table 1). Module-trait relationships analysis revealed that the eigenproteins of the brown module (68 proteins), turquoise module (100 proteins), green module (38 proteins), blue module (84 proteins), and yellow module (40 proteins) were associated with the total score of the CTQ (*r* = −0.15, *p* = 0.009); sampling time, antidepressant usage, and overeating item of the SCL-90-R (SCL-60) (*r* = 0.16, *p* = 0.006); antipsychotic usage, antidepressant usage, and total score of the YMRS (*r* = −0.15, *p* = 0.008); total score of the CTQ (*r* = −0.12, *p* = 0.03); and exercise (*r* = 0.12, *p* = 0.04), respectively (Fig. 2). Linear regression analysis revealed that the eigenprotein of the turquoise module remained statistically significant for the overeating item of the SCL-90-R after controlling for sampling time and antidepressant usage (*t* = 2.400, *p* = 0.017).

Additional analysis of hospital type using ANOVA revealed that only the green module was significantly associated with hospital type (*F* = 4.356, *p* = 0.001). After controlling for hospital type, the eigenprotein of the green module was no longer statistically significant with the total YMRS score (*t* = −1.804, *p* = 0.07).

Analysis was conducted with each individual item of the MADRS. The brown and turquoise modules did not exhibit statistical significance for any individual item of the MADRS. The green module exhibited a statistical significance for MADRS-4 (reduced sleep) and MADRS-6 (concentration difficulty), but no significant association was noted after controlling for hospital type.

After considering the strength of the associations between modules and traits, and as the green module was influenced by hospital type, further analysis was performed for the brown and turquoise modules.

### Hub protein identification of the brown and turquoise module

**Fig. 3:**
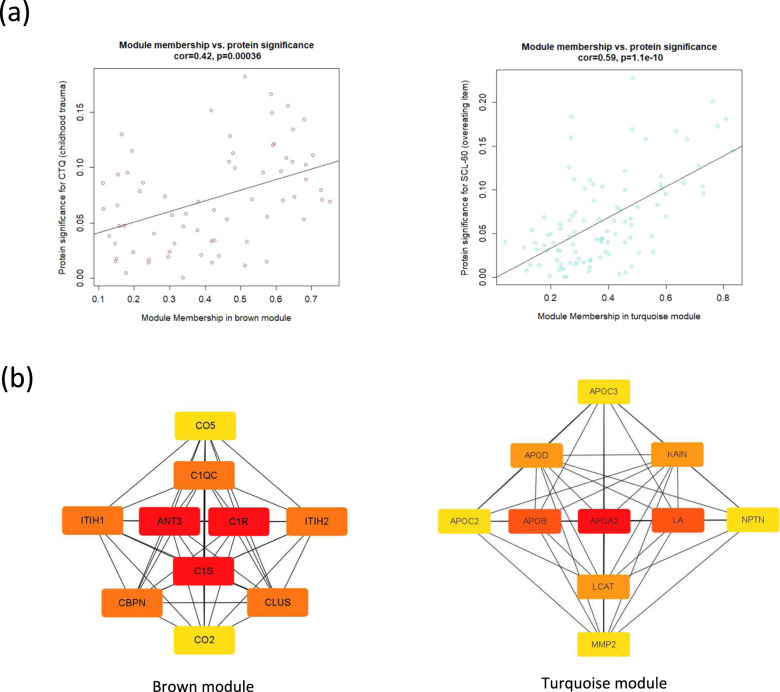
Significant proteins in individual protein modules. **a** Scatter plot between protein significance for childhood trauma (left) and overeating (right) and module membership in brown (left) and turquoise (right) modules, respectively. The correlation coefficient was calculated with Pearson’s correlation analysis. **b** The top 10 hub proteins of the brown module (left) and turquoise module (right) are presented.

In the brown module, module membership and protein significance for the total score of CTQ exhibited a significant linear relationship. In the turquoise module, module membership and protein significance for the overeating item of SCL-90-R (SCL-60) exhibited a significant linear relationship (Fig. 3a). The top 10 hub proteins in these modules based on MCC are illustrated in Fig. 3b, and the module membership for each protein is presented in Supplementary Table 2. Complement components and apolipoproteins were dominant in the brown and turquoise modules, respectively.

### Functional annotation of the brown and turquoise module

For the brown module, significant terms were related to immune responses. For the turquoise module, significant terms were predominantly related to lipid metabolism. The detailed terms and pathways of the functional analysis are summarized in Table 2, and more comprehensive results are presented in Supplementary Table 3.

**Table 2:** Functional analysis of significant protein modules.

	Brown module				Turquoise module			
	Function	Number of proteins	*P* value	*Q* value	Function	Number of proteins	*P* value	*Q* value
BP	Proteolysis	32	5.69E−15	1.31E−11	Plasma lipoprotein particle remodeling	8	1.14E−10	1.82E−07
	Protein activation cascade	14	2.47E−14	2.84E−11	Protein–lipid complex remodeling	8	1.14E−10	1.82E−07
	Complement activation	13	1.63E−13	1.25E−10	Macromolecular complex remodeling	8	1.86E−10	1.99E−07
	Humoral immune response	15	3.51E−11	2.01E−08	Plasma lipoprotein particle organization	8	2.80E−09	2.25E−06
	Response to external stimulus	32	2.72E−10	1.23E−07	Lipid transport	16	3.94E−09	2.36E−06
CC	Extracellular space	54	4.35E−37	1.30E−34	Extracellular region part	68	6.18E−22	2.42E−19
	Extracellular region part	58	1.37E−27	2.03E−25	High-density lipoprotein particle	13	3.87E−20	7.58E-18
	Blood microparticle	20	1.82E−24	1.81E−22	Extracellular space	49	1.18E−19	1.54E−17
	Extracellular region	58	4.06E−23	3.02E−21	Plasma lipoprotein particle	13	1.67E−18	1.31E−16
	Extracellular vesicle	40	3.72E−18	1.90E−16	Lipoprotein particle	13	1.67E−18	1.31E−16
MF	Serine-type endopeptidase inhibitor activity	10	2.25E−10	6.69E−08	Cell adhesion molecule binding	15	1.14E−06	4.73E−04
	Endopeptidase inhibitor activity	11	2.80E−09	3.78E−07	Receptor binding	26	5.02E−06	1.04E−03
	Peptidase inhibitor activity	11	3.99E−09	3.78E−07	Lipid binding	17	1.06E−05	1.46E−03
	Endopeptidase regulator activity	11	5.09E−09	3.78E−07	Enzyme inhibitor activity	12	1.54E−05	1.60E−03
	Peptidase regulator activity	11	3.02E−08	1.62E−06	Serine-type endopeptidase inhibitor activity	7	2.06E−05	1.71E−03
KEGG	Complement and coagulation cascades	20	1.61E−26	2.18E−24	Cholesterol metabolism	9	7.00E−09	1.16E−06

*BP* biological process, *CC* cellular component, *MF* molecular function, *KEGG* Kyoto Encyclopedia of Genes and Genomes.

The top five terms of DAVID functional annotation of BP, CC, MF, and the top KEGG pathway are presented.

*Q* value based on the Benjamini FDR correction.

### Module stability

Network stability analysis revealed that reproducible protein-module assignments had averages of 81.35% and 82.77% for proteins in the brown and turquoise modules, respectively (Supplementary Fig. 3).

## Discussion

In this study of individuals with affective disorders, weighted correlation network analysis revealed six protein modules (excluding the gray module) in which two protein network modules were significantly associated with childhood trauma and overeating. The brown module with childhood trauma and the turquoise module with overeating exhibited significant associations between module membership and protein significance. Key proteins and functions were identified, with the lack of any association between the differentiation of MDD and BD based on the constructed protein network modules.

Compared to other studies conducted using peripheral blood [9, 36], simultaneous multiplex quantification of numerous proteins enabled us to conduct an analysis using a larger number of proteins. In addition, the effects of age, sex, and BMI of protein masses were discarded to reduce the likelihood of identifying associations with factors other than psychopathology [37, 38], which was also in line with previous weighted correlation network analyses [39*–*42]. This approach enabled us to identify psychopathological traits associated with correlated network-based mechanisms. In this regard, an analysis using a single or few individual protein marker(s) seems insufficient to encompass complex biological pathways.

The brown module with 68 proteins was associated with the degree of childhood trauma. Several complement components were hub proteins within this network, alongside other proteins such as ITIHs and ANT3. Hub proteins with the highest connectivity were C1R, C1S, and ANT3. C1R and C1S are the proteases responsible for the activation and proteolytic activity of the C1 complex [43]. ANT3 is a serine protease inhibitor that suppresses coagulation and hemostasis, and inflammation [44]. The main roles of these proteins are to regulate complement and coagulation pathways, which are known to interact with each other in BD [45] and MDD [46]. In addition, there is evidence that these pathways are dysregulated even from childhood before patients develop psychotic symptoms [47]. Dysregulation is also observed in those with post-traumatic stress disorder [48], and there was a report that multiple traumatic events were directly proportional to the degree of systemic inflammation [49]. Our result is in line with these studies and proposes that key proteins that regulate complement and coagulation pathways are actually associated with childhood trauma. Although evidence of immune dysfunction and inflammation in adults with childhood trauma among individuals with affective disorders is accumulating [50], previous studies have focused on glucocorticoid pathways and interleukins [51*–*54]. Our study expands previous findings to network-level protein co-expression.

The presence of childhood trauma is known to increase the severity of these disorders and is associated with greater treatment resistance and a higher relapse rate [55*–*57]. A recent randomized control trial of infliximab for bipolar depression revealed that this treatment was effective in individuals with childhood trauma [58]. The present finding may explain why individuals with childhood trauma are resistant to conventional therapies. In this regard, agents with anti-inflammatory and immune function properties that target the co-expression of these proteins may be beneficial. Further investigation in a longitudinal design to determine the association between treatment-resistant depression and the co-expression of complement and coagulation pathways would be informative.

The turquoise module with 100 proteins was associated with overeating. Accumulating evidence based on genomics, transcriptomics, and proteomics suggests that individuals of MDD with hyperphagia have more dominant inflammatory-metabolic properties [59*–*61]. Our study expanded these findings to a transdiagnostic affective disorder population, including both MDD and BD. Furthermore, our results highlighted an interplay between these proteins rather than the involvement of individual proteins. When comparing hyperphagia-associated proteomic modules from our study with the NESDA study, APOB was the only common hub protein [9]. Our study removed the effects of age, sex, and BMI, and the proteins that were actually quantified had discrepancies, which would have yielded different networks. In our study, apolipoproteins were the major hub proteins of the hyperphagia-associated module.

The key protein with the highest connectivity in this module was APOA2 (Apolipoprotein A-II), which is the second most abundant apolipoprotein in high-density lipoprotein particles [62]. Its alteration is known to be involved in various conditions, including metabolic syndrome and insulin resistance [63]. Considering psychiatric studies, its level was associated with metabolic syndrome in schizophrenia [64], associated with psychotropic medication in suicide completers [65], and altered in the prodromal stage of BD [66]. These studies did not specifically evaluate appetite, and direct comparison is limited, but some results do suggest that a metabolic subtype could be involved. APOA2 is related to tryptophan metabolism, which is not only related to appetite but also to depression, as tryptophan is the main precursor of serotonin [67, 68].

Several studies have identified an association between affective disorders and individual apolipoprotein markers [69*–*71]. However, research with individual apolipoproteins in affective disorders has mainly focused on cognition [72, 73], and the association with hyperphagia warrants further investigation. Nevertheless, an association between atypical depression and lipid metabolism has been reported [74]. The current evidence of these individual associations may not reflect complex protein-level networks, underscoring the need for further network-level-based studies. Further analysis entangling the sophisticated association between apolipoproteins, appetite, and depression should be investigated in the future.

On the other hand, no module was associated with the differentiation of MDD and BD. Most of the proteins from our multi-protein model in our previous study that differentiated MDD and BD were dispersed in several protein modules [10]. As there are various mechanisms that are related to the differentiation of these disorders, they probably would not be captured in a single protein network. Additionally, this implies that without the consideration of childhood trauma and hyperphagia, the differentiation of MDD and BD based on circulating proteomics has limitations. The need to focus on the degree of childhood trauma and overeating may be required, not only in biological studies but also in clinical settings, as biological treatment responses could be affected by these protein networks. Interestingly, despite several controversies, both childhood trauma and hyperphagia have been proposed as risk factors for conversion from MDD to BD [75, 76]. Therefore, not only should we consider the presence of (hypo)manic symptoms, but also consider these clinical traits, or integrate these clinical traits with biological correlates, to precisely phenotype those with affective disorders [77, 78].

### Strengths and limitations

The following limitations of the study should be considered. First, the network was based on peripheral proteins; hence, the functional analysis has limitations in interpretation. Despite evidence of blood–brain barrier dysfunction in psychiatric disorders, peripheral blood does not always reflect the CNS. In this regard, our data suggest that childhood trauma and overeating in affective disorders are associated with systematic immune function and lipid metabolism. Second, other traits may not have been considered. Although various scales were utilized, other known factors that are known to help differentiate MDD from BD, including familial history [79] and treatment-resistant depression [80], need to be analyzed in the future. Third, there may have been other proteins that were not covered. However, the number of proteins quantified in this study was substantially larger than that in previous studies, and analysis was performed on a targeted basis method. Fourth, the interpretation of causality is limited due to the cross-sectional design. This study is based on correlation interference and not on causal interference. Longitudinal analysis with multiple measurements of clinical traits and plasma proteomes will enable investigation of the preservation of proteomic networks and their relationship with clinical traits. Fifth, sex-stratified analysis could not be interpreted due to decreased stability of the co-expression network. Although we discarded the effect of sex, it is known that males and females have distinct innate and adaptive responses [81]. A larger sample size could overcome this limitation in the future. Finally, independent validation was not performed in this study. However, subsampling was performed on the protein modules to reveal a rather stable network.

Nevertheless, to our knowledge, this study is the first to construct plasma protein networks with numerous proteins and to apply these networks to a transdiagnostic population of affective disorders. As proteins reflect biological functions, the degree of childhood trauma and overeating should be considered as candidates for endophenotypes in affective disorders. Further studies, including replication and longitudinal designs, will enable us to verify these results.

## Supplementary information

Supplementary Figure 1, 2, 3

Supplementary Table 1, 2, 3

## Data Availability

The raw MRM-MS files, including quantitative MS spectra for 454 peptides/420 proteins for all 299 plasma samples, were deposited into PeptideAtlas (Dataset identifier: PASS04825, Password: SA5392tsq). The other datasets presented in this study may be available from the corresponding authors upon reasonable request.

## References

[1] Kendler KS (2021). The characteristic signs and symptoms of mania and depression according to Kraepelin circa 1905: a comparison with DSM-III. Psychol Med.

[2] Qi YJ, Lu YR, Shi LG, Demmers JAA, Bezstarosti K, Rijkers E (2022). Distinct proteomic profiles in prefrontal subareas of elderly major depressive disorder and bipolar disorder patients. Transl Psychiatry.

[3] Rai S, Griffiths KR, Breukelaar IA, Barreiros AR, Chen W, Boyce P (2021). Default-mode and fronto-parietal network connectivity during rest distinguishes asymptomatic patients with bipolar disorder and major depressive disorder. Transl Psychiatry.

[4] Rhee SJ, Han D, Lee Y, Kim H, Lee J, Lee K (2020). Comparison of serum protein profiles between major depressive disorder and bipolar disorder. BMC Psychiatry.

[5] Shin D, Rhee SJ, Lee J, Yeo I, Do M, Joo EJ (2021). Quantitative proteomic approach for discriminating major depressive disorder and bipolar disorder by multiple reaction monitoring-mass spectrometry. J Proteome Res.

[6] Preece RL, Han SYS, Bahn S (2018). Proteomic approaches to identify blood-based biomarkers for depression and bipolar disorders. Expert Rev Proteom.

[7] Aebersold R, Mann M (2016). Mass-spectrometric exploration of proteome structure and function. Nature.

[8] Pei G, Chen L, Zhang W (2017). WGCNA application to proteomic and metabolomic data analysis. Methods Enzymol.

[9] van Haeringen M, Milaneschi Y, Lamers F, Penninx B, Jansen R (2023). Dissection of depression heterogeneity using proteomic clusters. Psychol Med.

[10] Shin D, Rhee SJ, Shin D, Joo EJ, Jung HY, Roh S (2022). Integrating proteomic and clinical data to discriminate major psychiatric disorders: applications for major depressive disorder, bipolar disorder, and schizophrenia. Clin Transl Med.

[11] Jabbi M, Arasappan D, Eickhoff SB, Strakowski SM, Nemeroff CB, Hofmann HA (2020). Neuro-transcriptomic signatures for mood disorder morbidity and suicide mortality. J Psychiatr Res.

[12] Najjar S, Pearlman DM, Alper K, Najjar A, Devinsky O (2013). Neuroinflammation and psychiatric illness. J Neuroinflamm.

[13] Garay-Baquero DJ, White CH, Walker NF, Tebruegge M, Schiff HF, Ugarte-Gil C (2020). Comprehensive plasma proteomic profiling reveals biomarkers for active tuberculosis. JCI Insight.

[14] Vora N, Kalagiri R, Mallett LH, Oh JH, Wajid U, Munir S (2019). Proteomics and metabolomics in pregnancy—an overview. Obstet Gynecol Surv.

[15] Kim Y, Kang UB, Kim S, Lee HB, Moon HG, Han W (2019). A validation study of a multiple reaction monitoring-based proteomic assay to diagnose breast cancer. J Breast Cancer.

[16] Dong W, Qiu C, Gong D, Jiang X, Liu W, Liu W (2019). Proteomics and bioinformatics approaches for the identification of plasma biomarkers to detect Parkinson’s disease. Exp Ther Med.

[17] Ryan KM, Glaviano A, O’Donovan SM, Kolshus E, Dunne R, Kavanagh A (2017). Electroconvulsive therapy modulates plasma pigment epithelium-derived factor in depression: a proteomics study. Transl Psychiatry.

[18] Noorbakhsh F, Aminian A, Power C (2015). Application of “Omics” technologies for diagnosis and pathogenesis of neurological infections. Curr Neurol Neurosci Rep.

[19] Jayanthi S, Buie S, Moore S, Herning RI, Better W, Wilson NM (2010). Heavy marijuana users show increased serum apolipoprotein C-III levels: evidence from proteomic analyses. Mol Psychiatry.

[20] World Health Organization (2010). Global Recommendations on Physical Activity for Health: World Health Organization. 26180873

[21] Hafkenscheid A (1991). Psychometric evaluation of a standardized and expanded Brief Psychiatric Rating Scale. Acta Psychiatr Scand.

[22] Young RC, Biggs JT, Ziegler VE, Meyer DA (1978). A rating scale for mania: reliability, validity and sensitivity. Br J Psychiatry.

[23] Montgomery SA, Asberg M (1979). A new depression scale designed to be sensitive to change. Br J Psychiatry.

[24] Hamilton M (1959). The assessment of anxiety states by rating. Br J Med Psychol.

[25] Derogatis LR. SCL-90-R: Administration, scoring & procedures manual-II for the (revised) version and other instruments of the psychopathology rating scale series. Clin Psychometr Res. 1992:1–16. https://www.scirp.org/(S(i43dyn45teexjx455qlt3d2q))/reference/ReferencesPapers.aspx?ReferenceID=115039.

[26] The WHOQOL Group. Development of the World Health Organization WHOQOL-BREF quality of life assessment. Psychol Med. 1998;28:551–8. 10.1017/s0033291798006667 9626712

[27] Bernstein DP, Fink L (1998). Childhood Trauma Questionnaire: A retrospective self-report manual.

[28] Ward MF, Wender PH, Reimherr FW (1993). The Wender Utah Rating Scale: an aid in the retrospective diagnosis of childhood attention deficit hyperactivity disorder. Am J Psychiatry.

[29] Smith CS, Reilly C, Midkiff K (1989). Evaluation of three circadian rhythm questionnaires with suggestions for an improved measure of morningness. J Appl Psychol.

[30] Rosenthal NE, Sack DA, Gillin JC, Lewy AJ, Goodwin FK, Davenport Y (1984). Seasonal affective disorder. A description of the syndrome and preliminary findings with light therapy. Arch Gen Psychiatry.

[31] Langfelder P, Horvath S (2008). WGCNA: an R package for weighted correlation network analysis. BMC Bioinforma.

[32] Chin CH, Chen SH, Wu HH, Ho CW, Ko MT, Lin CY (2014). cytoHubba: identifying hub objects and sub-networks from complex interactome. BMC Syst Biol.

[33] Shannon P, Markiel A, Ozier O, Baliga NS, Wang JT, Ramage D (2003). Cytoscape: a software environment for integrated models of biomolecular interaction networks. Genome Res.

[34] Huang da W, Sherman BT, Lempicki RA (2009). Systematic and integrative analysis of large gene lists using DAVID bioinformatics resources. Nat Protoc.

[35] Tanabe M, Kanehisa M. Using the KEGG database resource. Curr Protoc Bioinform. 2012;Chapter 1:Unit1.12. 10.1002/0471250953.bi0112s38 22700311

[36] Jeffries CD, Perkins DO, Fournier M, Do KQ, Cuenod M, Khadimallah I (2018). Networks of blood proteins in the neuroimmunology of schizophrenia. Transl Psychiatry.

[37] Beijers L, Wardenaar KJ, Bosker FJ, Lamers F, van Grootheest G, de Boer MK (2019). Biomarker-based subtyping of depression and anxiety disorders using Latent Class Analysis. A NESDA study. Psychol Med.

[38] Beijers L, van Loo HM, Romeijn JW, Lamers F, Schoevers RA, Wardenaar KJ (2020). Investigating data-driven biological subtypes of psychiatric disorders using specification-curve analysis. Psychol Med.

[39] Gudmundsdottir V, Pedersen HK, Mazzoni G, Allin KH, Artati A, Beulens JW (2020). Whole blood co-expression modules associate with metabolic traits and type 2 diabetes: an IMI-DIRECT study. Genome Med.

[40] Kim S, Hwang Y, Webster MJ, Lee D (2016). Differential activation of immune/inflammatory response-related co-expression modules in the hippocampus across the major psychiatric disorders. Mol Psychiatry.

[41] Wingo AP, Dammer EB, Breen MS, Logsdon BA, Duong DM, Troncosco JC (2019). Large-scale proteomic analysis of human brain identifies proteins associated with cognitive trajectory in advanced age. Nat Commun.

[42] Xu J, Bankov G, Kim M, Wretlind A, Lord J, Green R (2020). Integrated lipidomics and proteomics network analysis highlights lipid and immunity pathways associated with Alzheimer’s disease. Transl Neurodegener.

[43] Rossi V, Bally I, Lacroix M, Arlaud GJ, Thielens NM (2014). Classical complement pathway components C1r and C1s: purification from human serum and in recombinant form and functional characterization. Methods Mol Biol.

[44] Levy JH, Sniecinski RM, Welsby IJ, Levi M (2016). Antithrombin: anti-inflammatory properties and clinical applications. Thromb Haemost.

[45] Rodrigues JE, Martinho A, Santos V, Santa C, Madeira N, Martins MJ (2022). Systematic review and meta-analysis on MS-based proteomics applied to human peripheral fluids to assess potential biomarkers of bipolar disorder. Int J Mol Sci.

[46] Ruland T, Chan MK, Stocki P, Grosse L, Rothermundt M, Cooper JD (2016). Molecular serum signature of treatment resistant depression. Psychopharmacology.

[47] English JA, Lopez LM, O’Gorman A, Föcking M, Hryniewiecka M, Scaife C (2018). Blood-based protein changes in childhood are associated with increased risk for later psychotic disorder: evidence from a nested case-control study of the ALSPAC longitudinal birth cohort. Schizophr Bull.

[48] Speer K, Upton D, Semple S, McKune A (2018). Systemic low-grade inflammation in post-traumatic stress disorder: a systematic review. J Inflamm Res.

[49] Gola H, Engler H, Sommershof A, Adenauer H, Kolassa S, Schedlowski M (2013). Posttraumatic stress disorder is associated with an enhanced spontaneous production of pro-inflammatory cytokines by peripheral blood mononuclear cells. BMC Psychiatry.

[50] Renna ME, Peng J, Shrout MR, Madison AA, Andridge R, Alfano CM (2021). Childhood abuse histories predict steeper inflammatory trajectories across time. Brain Behav Immun.

[51] Müller N, Krause D, Barth R, Myint AM, Weidinger E, Stettinger W (2019). Childhood adversity and current stress are related to pro- and anti-inflammatory cytokines in major depression. J Affect Disord.

[52] Hellmann-Regen J, Spitzer C, Kuehl LK, Schultebraucks K, Otte C, Wingenfeld K (2019). Altered cellular immune reactivity in traumatized women with and without major depressive disorder. Psychoneuroendocrinology.

[53] Green C, Stolicyn A, Harris MA, Shen X, Romaniuk L, Barbu MC (2021). Hair glucocorticoids are associated with childhood adversity, depressive symptoms and reduced global and lobar grey matter in Generation Scotland. Transl Psychiatry.

[54] Iob E, Baldwin JR, Plomin R, Steptoe A (2021). Adverse childhood experiences, daytime salivary cortisol, and depressive symptoms in early adulthood: a longitudinal genetically informed twin study. Transl Psychiatry.

[55] Grillault Laroche D, Godin O, Belzeaux R, M’Bailara K, Loftus J, Courtet P (2022). Association between childhood maltreatment and the clinical course of bipolar disorders: a survival analysis of mood recurrences. Acta Psychiatr Scand.

[56] Opel N, Redlich R, Dohm K, Zaremba D, Goltermann J, Repple J (2019). Mediation of the influence of childhood maltreatment on depression relapse by cortical structure: a 2-year longitudinal observational study. Lancet Psychiatry.

[57] Nikkheslat N, McLaughlin AP, Hastings C, Zajkowska Z, Nettis MA, Mariani N (2020). Childhood trauma, HPA axis activity and antidepressant response in patients with depression. Brain Behav Immun.

[58] McIntyre RS, Subramaniapillai M, Lee Y, Pan Z, Carmona NE, Shekotikhina M (2019). Efficacy of adjunctive infliximab vs placebo in the treatment of adults with bipolar i/ii depression: a randomized clinical trial. JAMA Psychiatry.

[59] de Kluiver H, Jansen R, Milaneschi Y, Penninx B (2019). Involvement of inflammatory gene expression pathways in depressed patients with hyperphagia. Transl Psychiatry.

[60] Milaneschi Y, Lamers F, Peyrot WJ, Baune BT, Breen G, Dehghan A (2017). Genetic association of major depression with atypical features and obesity-related immunometabolic dysregulations. JAMA Psychiatry.

[61] Simmons WK, Burrows K, Avery JA, Kerr KL, Taylor A, Bodurka J (2020). Appetite changes reveal depression subgroups with distinct endocrine, metabolic, and immune states. Mol Psychiatry.

[62] Florea G, Tudorache IF, Fuior EV, Ionita R, Dumitrescu M, Fenyo IM (2022). Apolipoprotein A-II, a player in multiple processes and diseases. Biomedicines.

[63] Chan DC, Ng TW, Watts GF (2012). Apolipoprotein A-II: evaluating its significance in dyslipidaemia, insulin resistance, and atherosclerosis. Ann Med.

[64] Boiko AS, Mednova IA, Kornetova EG, Semke AV, Bokhan NA, Loonen AJM (2019). Apolipoprotein serum levels related to metabolic syndrome in patients with schizophrenia. Heliyon.

[65] Kim MJ, Do M, Han D, Son M, Shin D, Yeo I (2022). Proteomic profiling of postmortem prefrontal cortex tissue of suicide completers. Transl Psychiatry.

[66] Lee H, Han D, Rhee SJ, Kim J, Lee Y, Kim EY (2022). Alterations in blood proteins in the prodromal stage of bipolar II disorders. Sci Rep.

[67] Lai CQ, Smith CE, Parnell LD, Lee YC, Corella D, Hopkins P (2018). Epigenomics and metabolomics reveal the mechanism of the APOA2-saturated fat intake interaction affecting obesity. Am J Clin Nutr.

[68] Davidson M, Rashidi N, Nurgali K, Apostolopoulos V (2022). The role of tryptophan metabolites in neuropsychiatric disorders. Int J Mol Sci.

[69] Morris G, Berk M, Walder K, O’Neil A, Maes M, Puri BK (2021). The lipid paradox in neuroprogressive disorders: causes and consequences. Neurosci Biobehav Rev.

[70] So HC, Chau CK, Cheng YY, Sham PC (2021). Causal relationships between blood lipids and depression phenotypes: a Mendelian randomisation analysis. Psychol Med.

[71] Pomara N, Bruno D, Plaska CR, Ramos-Cejudo J, Osorio RS, Pillai A (2022). Plasma Amyloid-β dynamics in late-life major depression: a longitudinal study. Transl Psychiatry.

[72] Zhang SF, Chen HM, Xiong JN, Liu J, Xiong J, Xie JZ (2022). Comparison of cognitive impairments with lipid profiles and inflammatory biomarkers in unipolar and bipolar depression. J Psychiatr Res.

[73] Rhodes E, Insel PS, Butters MA, Morin R, Bickford D, Tosun D (2021). The impact of amyloid burden and APOE on rates of cognitive impairment in late life depression. J Alzheimers Dis.

[74] Lamers F, Vogelzangs N, Merikangas KR, de Jonge P, Beekman AT, Penninx BW (2013). Evidence for a differential role of HPA-axis function, inflammation and metabolic syndrome in melancholic versus atypical depression. Mol Psychiatry.

[75] Mitchell PB, Goodwin GM, Johnson GF, Hirschfeld RM (2008). Diagnostic guidelines for bipolar depression: a probabilistic approach. Bipolar Disord.

[76] Gilman SE, Dupuy JM, Perlis RH (2012). Risks for the transition from major depressive disorder to bipolar disorder in the National Epidemiologic Survey on Alcohol and Related Conditions. J Clin Psychiatry.

[77] Maes M, Moraes JB, Bonifacio KL, Barbosa DS, Vargas HO, Michelin AP (2021). Towards a new model and classification of mood disorders based on risk resilience, neuro-affective toxicity, staging, and phenome features using the nomothetic network psychiatry approach. Metab Brain Dis.

[78] Teicher MH, Gordon JB, Nemeroff CB (2022). Recognizing the importance of childhood maltreatment as a critical factor in psychiatric diagnoses, treatment, research, prevention, and education. Mol Psychiatry.

[79] Musliner KL, Østergaard SD (2018). Patterns and predictors of conversion to bipolar disorder in 91 587 individuals diagnosed with unipolar depression. Acta Psychiatr Scand.

[80] Bukh JD, Andersen PK, Kessing LV (2016). Rates and predictors of remission, recurrence and conversion to bipolar disorder after the first lifetime episode of depression-a prospective 5-year follow-up study. Psychol Med.

[81] Fish EN (2008). The X-files in immunity: sex-based differences predispose immune responses. Nat Rev Immunol.

