# Supplementary material for: Network analysis of plasma proteomes in affective disorders
Source: Transl Psychiatry. 2023 Jun 9;13:195. doi: 10.1038/s41398-023-02485-4 (PMC10256808; doi:10.1038/s41398-023-02485-4)
Supplement: Supplementary file 1 — Supplementary Figure 1, 2, 3 [file 41398_2023_2485_MOESM1_ESM.pptx]

## Slide 1
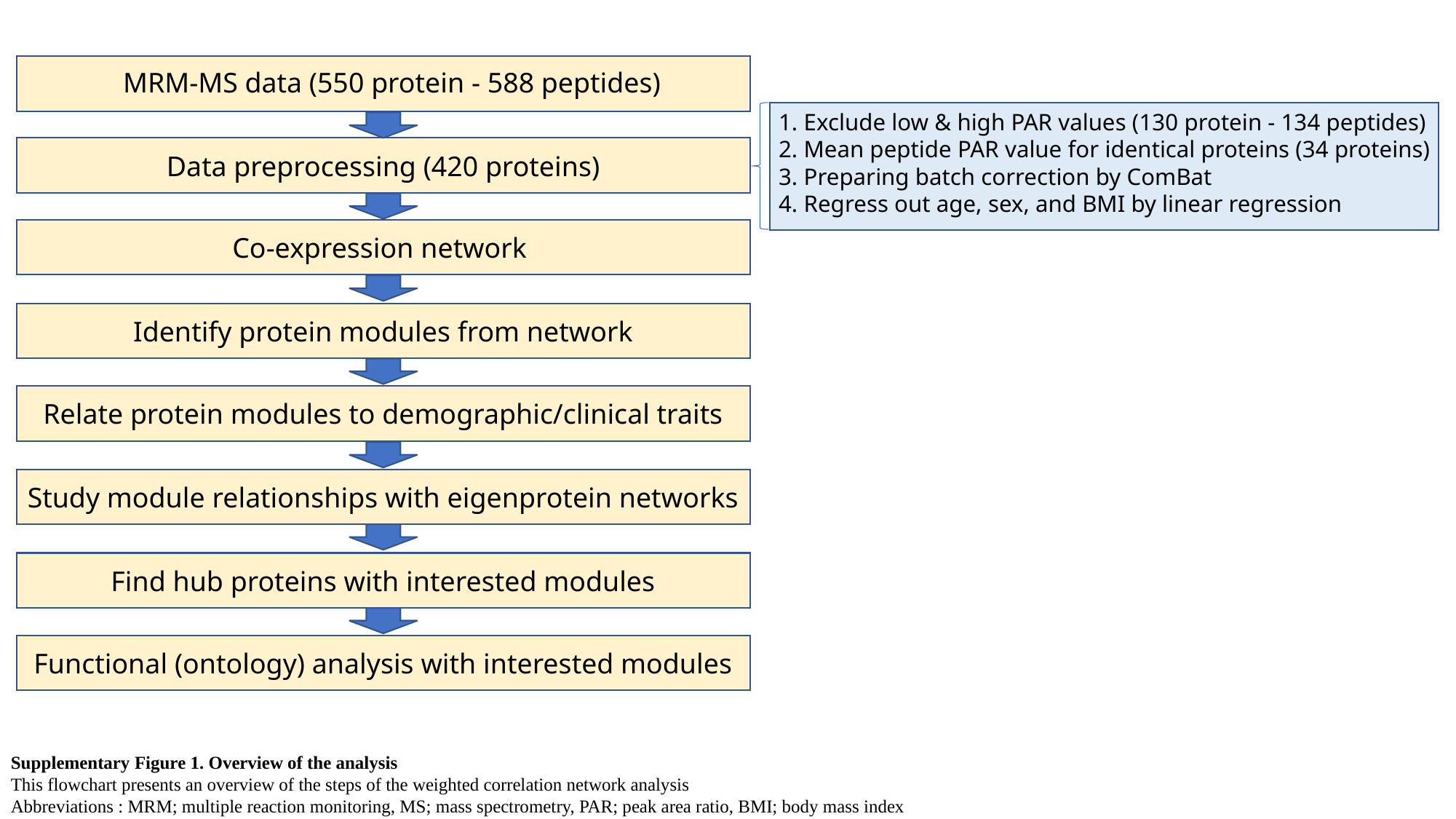

MRM-MS data (550 protein - 588 peptides)
1. Exclude low & high PAR values (130 protein - 134 peptides)
2. Mean peptide PAR value for identical proteins (34 proteins)
3. Preparing batch correction by ComBat
4. Regress out age, sex, and BMI by linear regression
Data preprocessing (420 proteins)
Co-expression network
Identify protein modules from network
Relate protein modules to demographic/clinical traits
Study module relationships with eigenprotein networks
Find hub proteins with interested modules
Functional (ontology) analysis with interested modules
Supplementary Figure 1. Overview of the analysis
This flowchart presents an overview of the steps of the weighted correlation network analysis
Abbreviations : MRM; multiple reaction monitoring, MS; mass spectrometry, PAR; peak area ratio, BMI; body mass index

## Slide 2
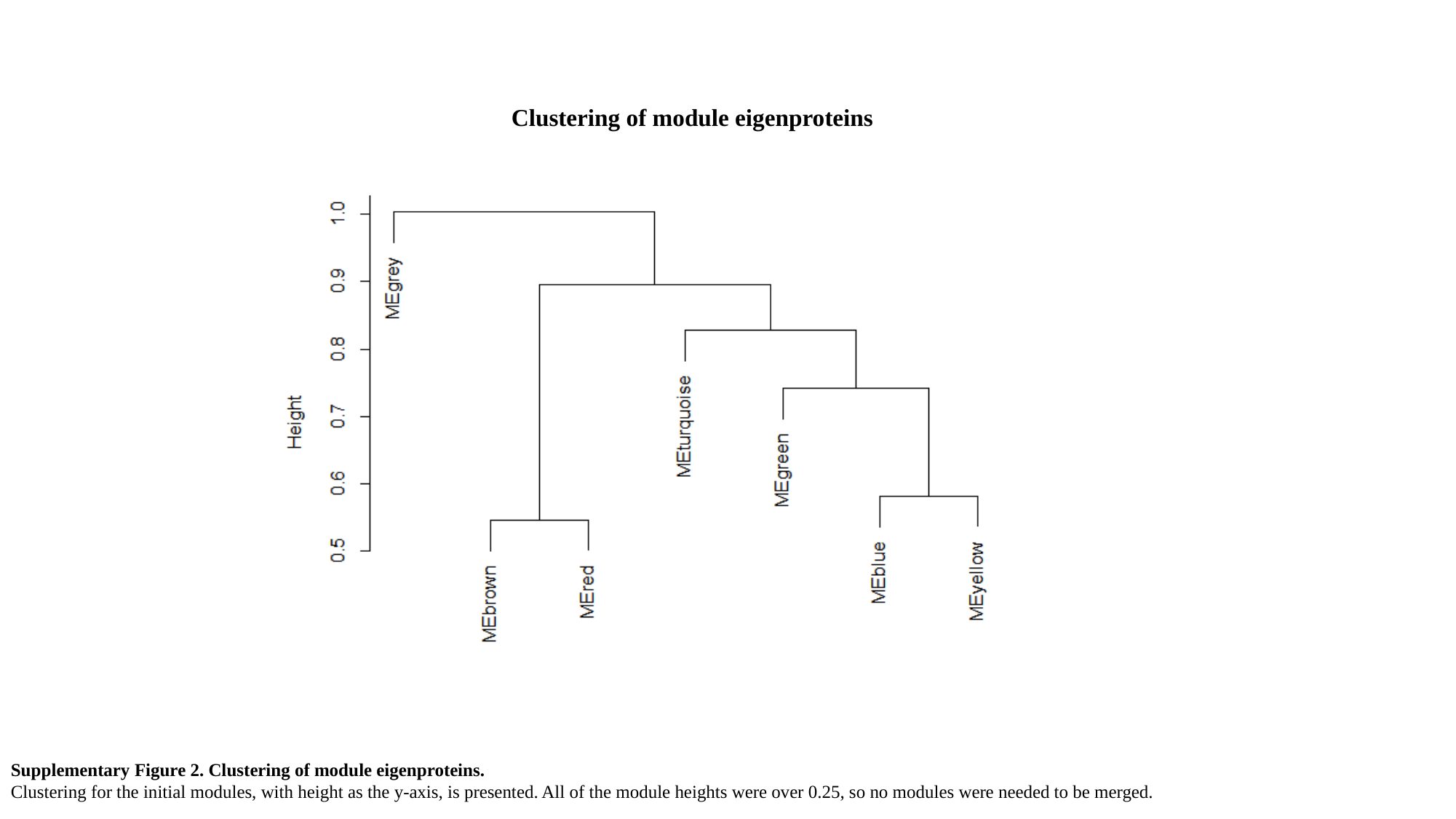

Clustering of module eigenproteins
Supplementary Figure 2. Clustering of module eigenproteins.
Clustering for the initial modules, with height as the y-axis, is presented. All of the module heights were over 0.25, so no modules were needed to be merged.

## Slide 3
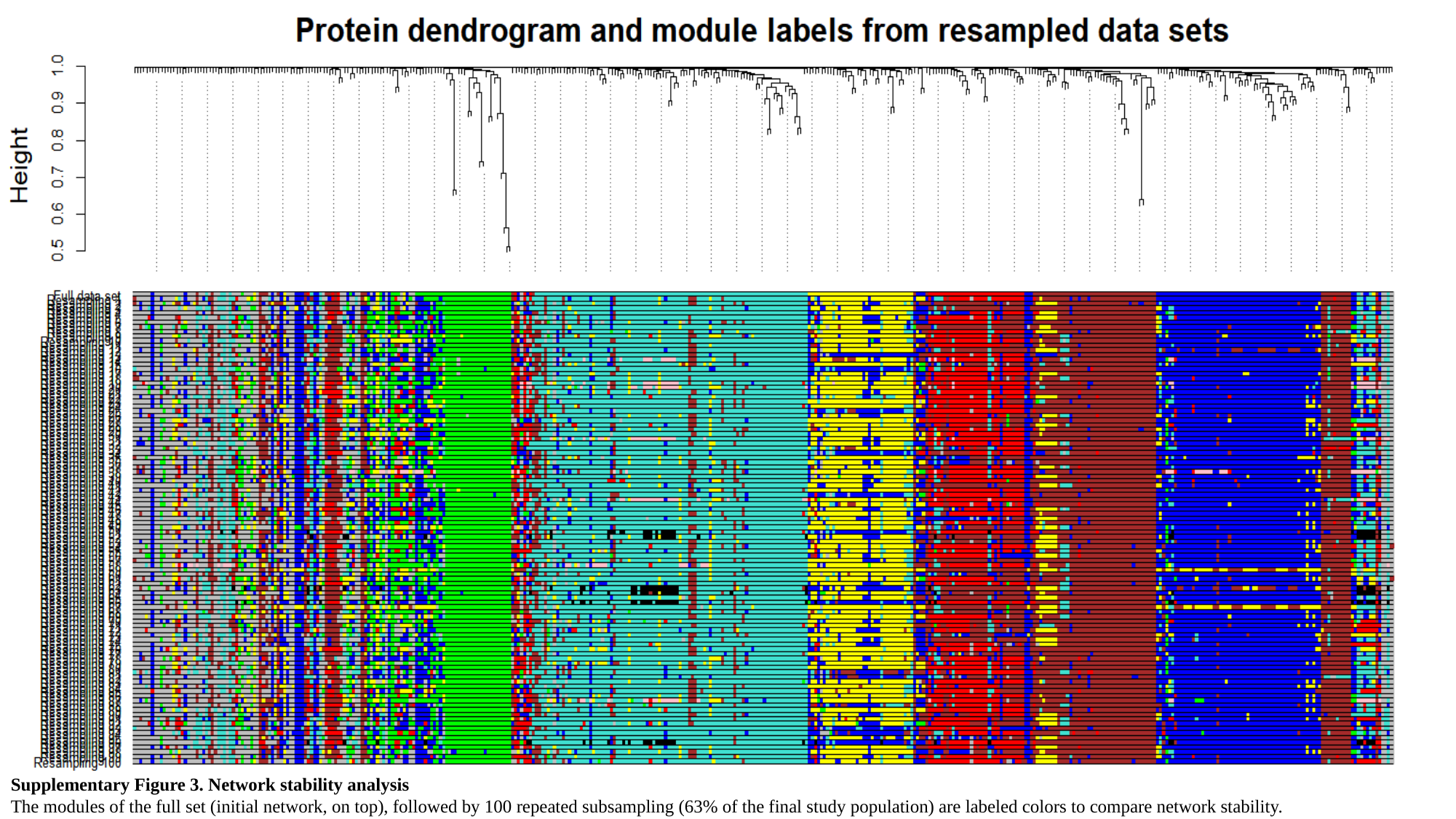

#
Supplementary Figure 3. Network stability analysis
The modules of the full set (initial network, on top), followed by 100 repeated subsampling (63% of the final study population) are labeled colors to compare network stability.
